# The Nursing Supplement

**Published:** 1888-05-26

**Authors:** 


### The Nursing Mirror.

All Contributions for this Supplement should be addressed "The Nursing Mirror," care of The Editor, 38, Old Jewry, E.C.

### En Passant.

NURSING IN AMERICA.—We asked a friend in America for some particulars of nursing work, but she refuses to give them. "I have been ill for two weeks," she writes from a New York hotel, "and during that time have had six trained nurses. I think the fact speaks for itself; do not ask me to go into particulars."

LADY DOCTORS IN GERMANY.—The German Women's Union has lately had a gift of eighty thousand marks for the purpose of promoting the study of medicine by women. It also expects another large gift towards building a gymnasium for girls. Mathilde Weber, of Bremen, in one of her latest works, declares that lady doctors are a moral and sanitary necessity for their own sex.

EALING COTTAGE HOSPITAL.—We have received from the Matron a report of the above hospital, which has lately been enlarged, and the nursing staff considerably added to. Miss Reid, who for many years did the greater part of the nursing, is now able to take more rest, and confine her labours to general superintendence. A hundred and fifty patients were treated last year, of whom 92 were discharged cured, 13 died, and 13 remained under treatment on December 31st.

NURSES ON STRIKE.—At the annual meeting last week of the Sheffield Nurses' Home, an institution for the provision of trained nurses, it appeared that owing to a dispute which had occurred between the committee and the lady superintendent, Miss Cowan, the latter had given three months' notice to resign her post, and the committee had since received a letter signed by thirty-one out of thirty-eight nurses connected with the Home, stating that unless Miss Cowan was asked to remain, and does remain, they will each tender their notices to leave at the earliest possible period. A resolution was passed approving the action of the committee, and the Mayor, who presided, said there was no need to close the Home because of the resignation of Miss Cowan or the nurses. They must consider Miss Cowan as dead when her notice expired, and act accordingly.

THE QUEEN'S NURSING FUND.—The Duke of Westminster has lately given the following particulars as to the disposal of the surplus of the Women's Jubilee offering to the Queen. There was in all about £70,000, which would for the first year yield an interest of £2,100, then an interest of £1,920 for fourteen years, and after that an interest of £1,750 a-year. It had been decided that this should be applied to the St. Katharine's Hospital, Regent's Park. Reviewing the history of this hospital during the time of its existence at St. Katharine's Docks, and after its removal to Regent's Park, the Duke said that part of the object of the founder seemed to have been to provide nurses, and there was much scope for development, much room for extended usefulness. The Queen was in favour of the idea, and it was possible that the very large property of the hospital, amounting probably to nearly £10,000 a-year, may be altogether given up to the object of a great central nursing home, making the institution a most valuable one.

METROPOLITAN NURSING ASSOCIATION.—Grosvenor House opened its doors once more last week for charitable purposes. The occasion was the annual meeting of the Metropolitan and National Nursing Association, which year by year is enlarging its work, and becoming more widely and better known. It now has branch Homes at Holloway, Paddington, Clapham, Hampstead, and Kensington, and also nurses working under local committees at Windsor, Westminster, Walworth, Bishop Auckland, Hertford, Hereford, Banbury, and Chelsea. The Duke of Westminster presided, and made a long and interesting speech; he was followed by Mr. Rathbone, who gave particulars of the cases nursed by the society and various other statistics. Mr. Kegan Paul also spoke, but the person whom the audience listened to with the greatest interest was Mr. Oscar Wilde. His speech was extremely laudatory; he praised the economy with which the society was managed, and the personal efforts of the nurses; and in conclusion he alluded with satisfaction to the great amount of charitable work done by women during the last century.

REMOVED TO THE HOSPITAL.—How often we come across this sentence in the daily papers, after an account of some accident or fire; and how little the outside public knows what it means from a nurse's point of view! Just when the ward is quiet and the lights turned down for the night, the doors swing open, and in comes a porter bearing a sweep who has been run over; or a child who has fallen in the fire. The sudden necessity for action disturbs the peace of the ward: a macintosh is thrown over a bed and the sweep there deposited, and then the nurse anxiously wonders how much soap and water she may safely apply. The patient has to be got to bed and all put straight before night nurse comes on duty; and the few quiet minutes which had been looked forward to are utterly lost. The child in its charred clothing, the woman with her throat cut, the man with delirium tremens; all cases which are beyond private resources, are "removed to the hospital," to be dealt with by doctors and nurses. We should like the readers of accounts of accidents to sometimes picture to themselves the scene when the victims reach the hospital, and to remember what a good thing it is there are such refuges for the seriously injured.

THE ZENANA MISSION.—We quote the following from an article entitled "Our Medical Work in Furredpore," which appeared in the small missionary magazine, *Our Indian Sisters*:—"It is very presumptuous to call it by such a name as medical work when we know so exceedingly little of medicine, but, nevertheless, the Lord has been helping and blessing us in this direction so much that we must let our friends know of it. Miss Gilbert had an annual grant of medicines for the treatment of women from the Government, and did considerable miscellaneous work, more specially among the very poor people, for whom no one cares but God. When she set out for England, she and we thought all the medicines would have to be corked up and left until she came back. But our dear Master meant us to do otherwise, for a few days after she started, our beloved brother Panchanon's wife became dangerously ill, and had to be brought to this house, as theirs was being rebuilt. She was not out of danger for more than a fortnight. Many nights I was called up to see our sister apparently dying, and to feel utterly at sea as to what treatment should be given. But always on asking the Lord the right medicines and nourishment were suggested, and our friend is now as well as ever." The article goes on to say how cholera broke out, and Mrs. Gilbert (who presumably knew nothing of medicine) being still away, the same treatment was pursued as in the case of brother Panchanon's wife; *i.e.*, a prayer was said, a medicine ignorantly chosen at random, and the poor patient dosed with it. Our respect for the Zenana Mission is fast vanishing.

### Original Articles.

### COUNTRY NURSES.

There are very few of our trained nurses who have any idea of the trials and troubles of a parish nurse in a rural district. The eccentricities and superstitions of country nursing are almost beyond belief, and the sturdy, independent farm labourer is a far more difficult patient to deal with than his town brother. To begin with, the parish is usually large and straggling, and in all weathers, at all hours of the day or night, the nurse will be expected to find her way along dark, muddy lanes, or to struggle across miles of lonely, storm-driven downs. A tricycle is a very great benefit to a country nurse; still, in the very bad weather, when it would be especially valuable, it is quite useless—a far more fatiguing means of progress than walking. However, by some means, a country nurse has often to cover from ten to fifteen miles a day, and luckily for her there are rare occasions when her register is empty and she can have a thorough rest. A sample of a day's work may be roughly sketched thus: While the nurse is enjoying her breakfast in her pretty little cottage parlour on a spring morning, a farmer rides up to the door to say that one of his labourers is very ill, and the doctor has been sent for, but could nurse go at once. The doctor lives in a neighbouring town three miles away, so that in cases of emergency the nurse is often sought first. Nurse hurries over the remainder of her breakfast, puts on her black hat and jacket (she has discarded the orthodox cloak and bonnet, finding them inconvenient), and carrying her basket, which always stands ready, she starts on her mission. A mile and a-half of pleasant walking across the fields in the fresh spring air, brings her to two small cottages, built of flints and thatched with straw. In one of these her patient lives, and nurse knocks at the door and enters. The usual sight of untidiness and disorder meets her view: two or three children are just being despatched to school by an elder sister; a bed is unmade in one corner of the room; and a baby in a crib is crying in another. In answer to a hasty question the elder girl directs nurse to enter the inner room, and here she finds the father and mother of the family: the first, a great strong man, now tossing restlessly on the bed, breathing rapidly and evidently very feverish; his wife hanging over him and trying to keep the bedclothes smooth. A few inquiries, and a few observations, and nurse is pretty sure that here is a bad case of pneumonia. There is no fire in the bed-room, and what is worse, no fireplace; it is therefore necessary to move the patient into the other room. The nurse hurries away the small children, quiets the baby by giving it a crust to suck, and then, with the help of the eldest girl, thoroughly cleans and tidies the room. The dirty crockery left from breakfast is banished to a small out-house which is used as a scullery; the accumulation of ashes in the fire-place is swept away, and a large log of wood thrown on the fire; the bed is well shaken and made up with clean sheets well aired before the fire, and then comes the chief difficulty of moving the patient. Luckily, the neighbour living in the next cottage is a great strong woman, and with her aid the removal is effected without any effort to the patient, who is partly delirious. Nurse has some Brand's Essence in her basket, and while she is making a cup of beef tea the doctor arrives. The nurse produces her case-book and all orders and remarks are entered; the wife is instructed what to do during the day, and the doctor requests nurse to come back and sit up with the patient for the night. In response to a query from the doctor why he was not sent for long before, there are the usual explanations of the long distance, no one to send, etc. The doctor shakes his head; he finds it very difficult to persuade these people to seek his aid till it is often too late to do any good. Great virtue is still set in many country places on various simples and home prescriptions. Poor people will go on trusting in nettle tea and Mother Smith's medicine till the near approach of death frightens them, and they are forced to send for skilled aid.

The doctor offers nurse a lift in his dog-cart to the next case, which is that of a small girl with croup and bronchitis. Brown Titus, her mother calls it, and she has with difficulty been persuaded that it is not good to hold the child head downwards when a fit of coughing comes on. The little patient looks much better to-day, and after a short visit the doctor hurries on his way, leaving nurse to go through the usual routine of bed making, washing, etc. Before he departs though, he takes nurse outside and asks her if she would mind calling at the vicarage about ten in the evening and giving the vicar's wife an injection of morphia. Nurse is proud to accept the trust, and with many promises to extreme care and caution she stows away the bottle and instrument handed to her in a safe corner of the basket. All the village knows that the vicar's wife is a confirmed invalid; but only a few know that the malady is cancer and there is no hope of recovery. Sometimes the poor lady has long spells of pain and suffering followed by sleeplessness, and it is no wonder if the doctor sometimes saves himself and his horse the long journey from the town on a dark night by trusting his hypodermic syringe into hands whose careful work he has watched for years. Nurse's next case is a publican's son suffering from abscesses. The boy has outgrown his strength, and is altogether in an unhealthy condition. What he needs is sea-air and quiet, but convalescent homes will not take him, and nurse has not yet heard of any refuge where she could send the poor sufferer. The sick room is over the bar, and all day long the carts are stopping and starting beneath the window, and in the evening the company often gets uproarious and the noise of vulgar songs and boorish laughter penetrates to the room above. After dressing the abscesses of this patient, nurse produces a book from the recesses of her basket, for this she has found is the best medicine for an irritable, unoccupied mind. Then nurse wends her way home, calling here and there to inquire after some former patient; and after she has had her dinner, she lies down for an hour or two preparatory to her night of watchfulness.

Such may be a specimen of work in a country parish, but it is a very commonplace and favourable one compared to the work on a stormy winter day, or in cottages where superstition reigns paramount. There are some ironworks in the Midlands, where when a man is choked by the heat and gas from the furnaces, his comrades dig a hole in the earth and put his head in it. The idea seems to be that the moist emanations of the fresh dug earth are so exactly opposite to the fierce, dry heat of the furnace that the contrast must be good. Then there is the old custom of the groaning cake, which is still cooked in many cottages on the birth of a child; and all who come to inquire after the mother are expected to eat a piece of the cake. Again, you will hear that a boy was born with a frog's mouth, because one of those animals was thrown at his mother shortly before her confinement. Probably the poor boy suffers from cleft palate, but all his life it will certainly be said that he has a frog's mouth. Another somewhat similar case was that of a girl born with a large mole on her arm, in which some person saw a resemblance to a mouse. Immediately the mother remembered that a few days before she had been startled when putting her hand into the cupboard, by a mouse running along the shelf.

These are only a few of the superstitions which prevail in country places, but it can easily be seen how hard a nurse's work must be in the face of such gross ignorance. Downright denial of the stories told must never be indulged in, or the cottagers take offence and will not suffer the presence of the nurse. Indeed, it needs quite as much tact as skill to nurse with success in the country.

### A Nurse's Note-book.

THE following are jottings taken in a women's medical ward:

No. 10. Received April 1st; aged twenty-four; three children; paralysis.—Very emaciated, and weak physically and mentally. The muscles of the throat are slightly paralysed, and she swallows with difficulty. Numerous bed-sores. Ordered to syringe sores, and dress them twice daily with antiseptic poultices. April 24th. Gradually getting worse; pulse 68 deg. Can no longer swallow solid food, and does not understand when spoken to. April 29th. Completely paralysed, and quite unconscious. Taking no nourishment, and sinking fast. May 1st. Died last night, after thirty-one days in hospital.

No. 7. Received April 4th; aged twenty; unmarried; acute rheumatism.—Cannot move hands or feet, and suffering acute pain. Affected joints to be bathed in soda and water, and packed in cotton wool every morning. Temperature 102 deg. April 27th. Patient did well till to-day, and was apparently getting better, when the temperature, pulse, and respirations all increased: due to pneumonia of left lung Dr. D. says. Poultices to be applied. May 2nd. Pneumonia better; rheumatism very bad in knee and ankle joints again. Temperature 100 degrees at night. May 5th. Patient had a sudden paroxysm of breathlessness; heart evidently affected. Not allowed to move hand or foot. Sister gave morphia injection at night. Limbs still packed in cotton wool. Temperature 99 degrees. May 8th. Further attacks of heart trouble, but not so severe as the first. Rheumatic pains less, temperature normal. The left leg is swollen, and slightly discoloured. Patient to be kept perfectly quiet and carefully watched. May 30. The paroxysms of breathlessness have ceased. Rheumatic pains also gone, but left leg still swollen and white; a bandage to be firmly applied from foot to hip twice a-day. May 28th. Patient moved on to the couch for the first time. June 5th. Discharged convalescent after sixty-two days in hospital.

No. 9. Received May 2nd; aged thirty; married; two children; diphtheria.—A strong-looking woman, but very ill when admitted. Put in tent near fire with steam kettle going. Inhalation every hour, throat mopped out every three hours, spray and poultice every four hours. Temperature to be taken every four hours; it ranges from 100 degrees upwards. May 8th. Patient still very ill and getting weak; can scarcely swallow. Temperature lower. May 16th. The throat is much better, and the inhaling, &c., is stopped. Weakness is increasing; no appetite. May 19th. No signs of improvement in the state of the throat; patient very weak and querulous. May 22nd. Died after twenty days in hospital.

No. 6. Received April 14th; aged eight; ulceration of mouth.—The child seems really to be suffering from starvation, and is on special diet. She is most fearfully emaciated, and does nothing but doze. Mouth to be painted every three hours with borax and honey. April 20th. Patient much better, and able to get up for an hour in the evening. She is the pet of the ward, but still very quiet and weak. May 5th. Child quite recovered so far as the mouth is concerned, but still not getting back her strength properly. May 8th. Removed to a convalescent home after twenty-four days in hospital.

No. 11. Received April 14; aged sixteen; typhoid fever.—The girl has been ill for ten days, is slightly delirious, and has a small bed-sore. Ordered to give beef-tea and milk every two hours; temperature to be taken every four hours. It is most difficult to dress the sore as the patient cannot be moved. April 25th. The usual day of the crisis has passed, and our poor patient seems no better. Always delirious, hands waving in the air, muttering constantly, and sometimes crying out. Nourishment every half-hour; injections are ordered. The sore is worse, it is almost impossible to attend to it. April 28th. Got slowly worse since Tuesday, and died early this morning, after fourteen days in hospital.

No. 2. Received April 24th; aged thirty-seven; married; six children; double pneumonia.—Jacket poultices every four hours, temperature taken every four hours. Mouth to be cleansed with Condy. May 1st. Temperature suddenly sunk to normal after a long sleep and profuse perspiration. I think the crisis is well passed. May 20th. Patient has slowly regained her strength, and was discharged convalescent to-day, after twenty-six days in hospital.

### Everybody's Opinion.

### HAPPILY A POPULAR DELUSION!

REPLY to Nurse Gertrude's experiences:—

Knowing a little about the Irish, their good nature and hospitality, I beg to ask Nurse Gertrude if she has not exaggerated in her description of the Kerry people. When they could afford to pay a nurse they cannot belong to the very poorer class; so that I should like to know if the food she describes as bad was unfit to eat. Nurses in hospitals (I know) often complain of their food. No doubt they locked her up to make her finish the work that they required, and considered it her duty to perform. As to the second item, I think the butler that offered (with a wink) champagne for a kiss, must entertain a very poor opinion of the nurse; and I would advise Nurse Gertrude to gain a little common sense, and put something more intelligent before the public next time. MANY YEARS A NURSE.

NURSE NANCY:—

Was there anything more in being offered a glass of wine at dinner than a glass of beer? Perhaps the old lady acted from experience when she put the bottle behind the door. I know many good nurses who always have a glass of beer to dinner and another to supper. As to myself, I always left it to others to sound my praise, and I advise Nurse Nancy to do the same. ALSO FOR MANY YEARS A NURSE.

### LET EXPERIENCE SPEAK

THE "Philanthropist" for May makes mention of a subject on which, through the kindness of THE HOSPITAL, I have once or twice been allowed to write, viz., the long hours and small pay of nurses—two things about which there should be no trouble were hospitals properly supported. It is no use asking "why do women go in for such badly-paid, such hard work"? Simply because we must work, there are so many thousands of us who work to live. Do the people who talk so much about the charms of hospital wards and patients, ever think what from half-past six a.m. to nine p.m. means to nurses for years together, with scant holiday now and again, with little chance of breathing the country air, with work that must be done, be the nurse ever so weary? Doctors are not famed for their consideration, and expect nurses to be pretty nearly perfect, whatever they may be themselves. And the staff is usually too small, and funds too low, to admit of more extra help than is absolutely necessary. Surely aid, they think—more precious gifts than a few flowers, a few old books and clothes, for which the donors have no use—would be forthcoming. Why will outsiders talk so much and do so little. Cooks, housemaids, nay even kitchenmaids, are better paid, and do less work than nurses; but we only complain of overwork, and of that plenty can be seen any day, if a romantic, gushing young lady would change places for a week with a hospital nurse. We are all but powerless to help ourselves; help must come from "outsiders" in good round yearly sums, so that a little extra help can be given when required, without the Lady Superintendent dreading the usual remark on pay day: "You appear to have a good many extra helpers, how is that?" There must be something very wrong in our Christianity when hospitals and convalescent homes languish so for want of funds. A. E. S.

### Lectures.

The last of the Thursday lectures at the Parkes Museum was delivered on May 17th by Mr. Lewis Tomalin, on "Healthy Clothing." After speaking of the functions of the skin, and the necessity for remembering that the human body is not a wooden dummy to be clothed without any regard for its nature and needs, the lecturer went on to explain the great difference between plant and animal fibre as used for clothing. Linen, and especially starched linen, was stigmatised as little better than macintosh, owing to its non-porous qualities; and even woollen garments which are lined with linen or cotton are very unhealthy from every point of view. Jaeger's sanitary clothing was highly recommended by Mr. Tomalin, as it is entirely composed of wool, is extremely porous, is undyed, and does not shrink. The clothing should be double down the front line of the body, as the blood-vessels terminate there, and should be buttoned on the shoulder to secure freedom from draughts. With regard to boots, Mr. Tomalin said that many people suffered a martyrdom with their feet, owing to the impervious manner in which they were covered. Stockings should be of pure wool, free from dye, and made like gloves with a separate partition for each toe. Boots can have a golosh sole, but the uppers must be of a porous woollen material. Certainly nurses should take this hint with regard to the covering of the feet. The lecturer condemned the linen collar as the giver of innumerable sore throats; he also strongly advised women to wear woollen corsets; for this garment is never changed so frequently as the rest of the underclothing, and if made of plant fibre, retains the exhalations of the skin. Woollen diapers for children were recommended, as less liable to give a chill when they remained damp. Clothing for night, as well as for day, was spoken about, linen sheets are to give place to blankets; and pillow-cases, mattresses and all, are to be entirely composed of animal fibre; the one thing desirable in clothing being perviousness and warmth. The lecturer wore a woollen collar, and (in spite of a wet day) porous boots.

### Exchange and Mart.

Under this heading we propose to insert purely Exchange advertisements, at a charge of sixpence for eighteen words, or three insertions for one shilling It must be distinctly understood that ordinary advertisements will not be inserted here.

RENSHAWS MANUALS. — "Anatomy," Malgaine (12s 6d.), "Elementary Botany, Dr. Silver (12s. 6d.), "Human Osteology" Ward (7s.) Would exchange either for Nurse's Surgical dressing case. What offer? Also Owen's "Materia Medica," "A Guide to the New Pharmacopœia" "First Book of Botany, Balfour. The last three for 5s.; or one guinea the lot. All in good condition. Address C., The Hospital Office, 38, Old Jewry, E.C.

### Appointments.

Miss C. Westlake, late of the Seamen's Hospital, Greenwich, has been appointed matron of the Blackheath and Charlton Cottage Hospital, in place of Mrs. Smith, resigned.

Miss Mary Pratt has been appointed matron of the Derbyshire General Infirmary after an exciting contest. Miss Pratt was trained at St. Thomas's Hospital, held the post of sister for some years in a London hospital, and was then appointed matron of Cardiff Infirmary, where she has laboured well for the last six years. At Cardiff Miss Pratt has been instrumental in establishing a training school for nurses, and a private nursing institution. At a largely-attended meeting of the governors, held last Friday in the board-room, Miss Pratt's resignation was received with deep regret, all feeling that her removal from Cardiff would be a serious loss to the infirmary.

### Vacancies.

The following vacancies are announced:—

**Matrons.**

Children's Hospital, Bradford; £50.
Broomhill Home, Kirkintilloch; £60.
Harlington Cottage Hospital; £30.
County Hospital, Dorchester; £60.

**Assistant Matron.**

Sister Dora Convalescent Hospital. Milford, near Stafford.

**Nurses.**

Bradford Eye Hospital.

Barrow-in-Furness; £20.

Bolton Infirmary; £25.

Guildford Hospital; £25.

Middlesex Industrial School, Feltham; 22s. per week.

Birkdale Home; several.

Royal Albert Hospital, Devonport, £25.

Newark Nurses' Institute; £20.

Children's Hospital, Cardington.

252, St. Paul's Road, Highbury; several.

**District Nurses.**

Miss Hunt's Home, Dublin.

Chester Deaconess Institution.

East London Nursing Society.

### Notes and Queries.

To Correspondents.—1. Questions may be written on post-cards. 2. Advertisements in disguise are inadmissible. 3. In answering a query please quote the number. 4. If a private answer is desired, a stamped addressed envelope must be forwarded.

16. *SisterE.* writes: I am a trained lady nurse, certified C.L., monthly, and I should be so very glad to fill up gaps for nurses from time to time if all expenses were paid. But quite how to make this known in your most valuable paper I do not quite see. I ought to say that I know surgical and medical work, and also have a knowledge of mental cases. I am 38.—I am, sir, faithfully yours, Sister E.

[We should be glad to facilitate any arrangements which readers may wish to make in response to Sister E's generous offer.—Ed.]

17. Would you kindly inquire through your valuable paper if there is any institution where a nurse could be trained in midwifery and monthly nursing free of charge?
Harraby. Trained Nurse.

**Answers.**

*Nurse Sarah* is requested to send name and address, not necessarily for publication. No reply can be given to anonymous communications.

